# Nature and Dynamics of Active Sites in Cu-Based Catalysts for the CO_2_ Hydrogenation to Methanol

**DOI:** 10.1021/acs.accounts.5c00599

**Published:** 2025-12-24

**Authors:** Aleix Comas-Vives, Christophe Copéret

**Affiliations:** † Institute of Materials Chemistry, TU Wien, 1060 Vienna, Austria; ‡ Department of Chemistry and Applied Biosciences, 27219 ETH Zurich, Vladimir-Prelog-Weg 2/10, CH-8093 Zurich, Switzerland

## Abstract

Catalytic CO_2_ hydrogenation to methanol is among the most attractive routes in CO_2_ conversion, as methanol is a chemical feedstock and a relevant energy carrier for the sustainable methanol economy. Cu-based catalysts are the typical choice for this reaction, and Cu-ZnO-Al_2_O_3_, the industrial reference material for hydrogenating CO (in the presence of CO_2_), has also been shown to perform well for CO_2_ hydrogenation. Adding other elements to Cu NPs as promoters (Zn, Ga, In, etc.) and using specific supports (ZrO_2_, Al_2_O_3_) enhance the catalytic activity and selectivity of Cu toward methanol, often minimizing the undesired competitive Reverse Water–Gas Shift and methanation reactions. However, these materials are complex, showing a delicate interplay between metal–metal and metal–support interactions in driving the overall selectivity toward methanol. Besides, the reactive gas-phase atmosphere (CO/CO_2_/H_2_/H_2_O in various ratios), in other words, the chemical potential, significantly affects the catalyst states, in terms of both structures and dynamics; this additional complexity often precludes the identification of the active sites, hampering the design of better catalytic materials based on structure–activity relationships derived from simple descriptors.

In this Account, we show how combining experiments and atomistic calculations provides detailed information on how interfaces, alloying, and dynamics play a crucial role in CO_2_ hydrogenation by stabilizing specific adsorbates from CO_2_ to key reaction intermediates, i.e., formate or methoxy. Specifically, we discuss the role played by metal/oxide interfaces and alloying/dealloying processes in driving catalytic activity (and selectivity); we also highlight how reaction conditions that define the chemical potential alter the stability and dynamics of the reactive states of catalysts. All of these aspects are crucial and interconnected, hence a challenge for both experimental and theoretical approaches.

This Account discusses these challenges and exemplifies their importance, focusing on the following: (i) How benchmarking catalytic models against experimental data is crucial in obtaining reliable computational models of the active sites; (ii) How specific surfaces/interfaces are particularly suited to stabilize key catalytic intermediates such as activated CO_2_, formate, and methoxy species; (iii) How dynamic changes in the systems can be accounted for via ab initio molecular dynamics combined with metadynamics, confronted with in situ X-ray absorption spectroscopy; (iv) How the “oxygen chemical potential” defined by the applied reaction conditions (e.g., H_2_/CO_2_ ratio) may affect the nature and stability of catalysts by using ab initio atomistic thermodynamics.

Finally, we provide an outlook on ongoing methodological developments that are needed to refine our understanding of the properties of these fascinating and dynamic materials.

## Graphical abstract

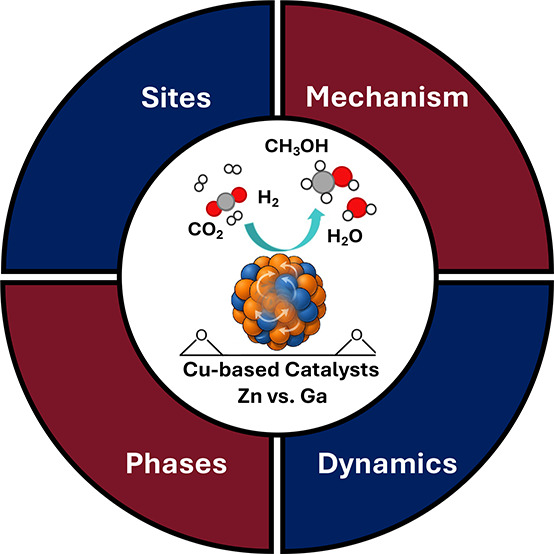

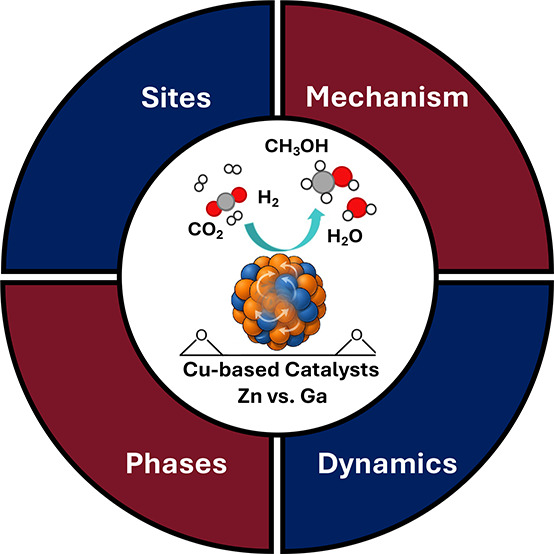

## Key References

-

Larmier, K. ; Liao, W. ; Tada, S. ; Lam, E. ; Verel, R. ; Bansode, A. ; Urakawa, A. ; Comas-Vives, A. ; Copéret, C. CO_2_-to-Methanol Hydrogenation on Zirconia-Supported Copper Nanoparticles: Reaction Intermediates and the Role of the Metal-Support Interface. Angew. Chem., Int. Ed. 2017, 56 (9), 2318–2323 10.1002/anie.20161016628111850.^1^ Spectroscopic measurements and DFT calculations identified formate and methoxy as key reaction intermediates and showed that the Cu/ZrO_2_ interface drives the CO_2_ conversion to methanol.
-

Lam, E. ; Corral-Pérez, J. J. ; Larmier, K. ; Noh, G. ; Wolf, P. ; Comas-Vives, A. ; Urakawa, A. ; Copéret, C. CO_2_ Hydrogenation on Cu/Al_2_O_3_: Role of the Metal/Support Interface in Driving Activity and Selectivity of a Bifunctional Catalyst. Angew. Chem., Int. Ed. 2019, 58 (39), 13989–13996 10.1002/anie.20190806031328855.^2^ Experiment and theory showed that Cu/γ-Al_2_O_3_ is bifunctional, with interfacial Al sites promoting CH_3_OH or CO formation and the Lewis acidic support favoring dimethyl ether and CO.
-

Zhou, H. ; Chen, Z. ; López, A. V. ; López, E. D. ; Lam, E. ; Tsoukalou, A. ; Willinger, E. ; Kuznetsov, D. A. ; Mance, D. ; Kierzkowska, A. ; Donat, F. ; Abdala, P. M. ; Comas-Vives, A. ; Copéret, C. ; Fedorov, A. ; Müller, C. R. Engineering the Cu/Mo_2_CT_*x*_ (MXene) Interface to Drive CO_2_ Hydrogenation to Methanol. Nat. Catal. 2021, 4 (10), 860–871 .^3^ Single-atom Cu on Mo_2_CT_*x*_ exhibited a δ^+^ nature that enhanced its catalytic activity in methanol synthesis and opened new reactive paths, where the Cu/Mo_2_CT_*x*_ interface played a key role.
-

Müller, A. ; Comas-Vives, A. ; Copéret, C. Ga and Zn Increase the Oxygen Affinity of Cu-Based Catalysts for the CO_*x*_ Hydrogenation According to Ab Initio Atomistic Thermodynamics. Chem. Sci. 2022, 13 (45), 13442–13458 36507169 10.1039/d2sc03107hPMC9685501.^4^ First-principles thermodynamics under CO_2_ hydrogenation conditions predicted that CuGa dealloys to Cu/GaO_*x*_ whereas CuZn only partially dealloys to Cu/ZnO_*x*_.

## 1 Introduction

Reducing the high CO_2_ atmospheric concentration has triggered a growing interest in developing sustainable catalytic processes to convert CO_2_ to fuels and chemicals.^5–7^ Methanol is foreseen as one of the key possible targets among the high-energy-density C_*n*_H_*y*_O_*z*_ compounds^8–11^ in the so-called methanol economy.^12^ Methanol is an easily transportable liquid at room temperature, which can be used directly as fuel or raw material to access key chemical feedstocks (through reforming,^13^ methanol-to-olefin (MTO),^14,15^ or Cativa processes^16^). Methanol is a possible product of CO_2_ hydrogenation, a thermodynamically favored reaction at low temperatures, which is foreseen to curb and mitigate CO_2_ emission, when using green H_2_ from renewable energy sources (eq 1). In fact, after the construction of the first plant in Iceland in 2012 by Carbon Recycling International (CRI),^17^ the CO_2_-to-renewable-methanol plant in Shunli (China) is the largest facility to produce fuel from captured CO_2_ emissions, with a yearly capacity of 110,000 tons of low-carbon methanol from 160,000 tons of CO_2_.^18^

Cu-based materials are among the most prominent catalysts because they show intrinsically low methane selectivity despite the highly thermodynamically favorable Sabatier process (methanation, eq 2).^19^ However, since the hydrogenation of CO_2_ requires high temperature (>200 °C), the Reverse Water–Gas Shift (RWGS) reaction (eq 3) becomes competitive, producing CO instead.

1 $$CO_{2}+3H_{2}↔CH_{3}\mathrm{OH}+H_{2}O,\Delta_{r}H^{{}^{\circ}}\left(500K\right)=-62\mathrm{kJ}{\mathrm{mol}}^{-1}$$

2 $$CO_{2}+4H_{2}↔CH_{4}+2H_{2}O,\Delta_{r}H^{{}^{\circ}}\left(500K\right)=-165\mathrm{kJ}{\mathrm{mol}}^{-1}$$

$$CO_{2}+H_{2}↔CO+H_{2}O,\Delta_{r}H^{{}^{\circ}}\left(500K\right)=+40\mathrm{kJ}{\mathrm{mol}}^{-1}$$ 3

Since silica is (mostly) an innocent support, Cu nanoparticles (NPs) supported on SiO_2_ can be used as a benchmark for Cu-based catalysts (Figure 1), although surface silanol density may affect catalytic activity,^20^ metal anchoring, and dispersion.^21^ In some systems, Cu–O–SiO_*x*_ interfacial bonds are present^22^ whereas strong metal–support interactions have also been suggested for Cu NPs on 2D-SiO_2_,^23^ with potential impact on the catalytic behavior.

**Figure 1:**
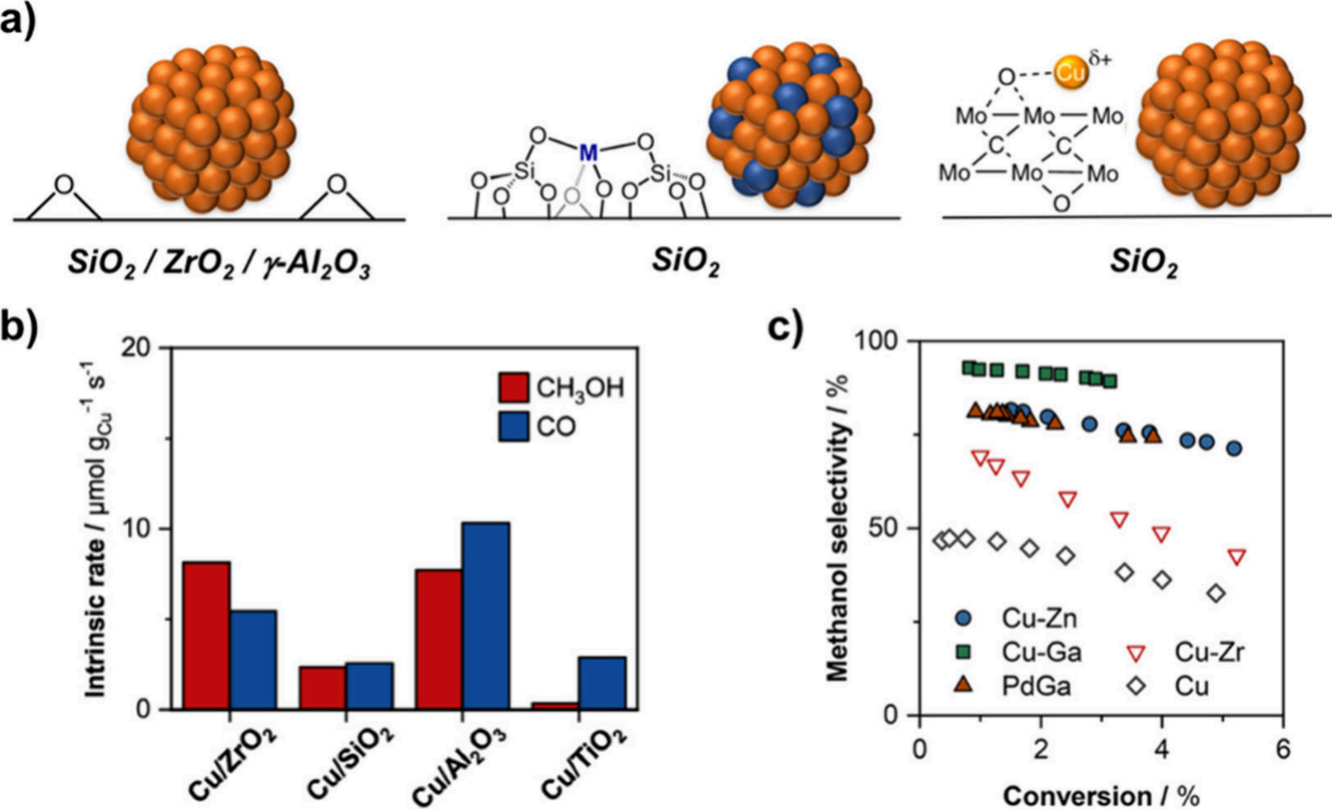
(a) Cu-based systems active in the CO_2_ conversion to methanol that are relevant to this Account. (b) Intrinsic rates of formation for Cu supported on different metal oxides, tested in identical conditions (3:1:1 H_2_/CO_2_/Ar, 25 bar, 230 °C, 5 g of SiC, 6–100 sccm). (c) Selectivity vs conversion (with reference materials Cu/Zr@SiO_2_ and Cu/SiO_2_) tested under identical conditions (3:1:1 H_2_/CO_2_/Ar, 25 bar, 230 °C, 5 g of SiC, 6–100 sccm).

Our reported Cu/SiO_2_ system shows low intrinsic activity and selectivity toward methanol (ca. 50:50 CH_3_OH/CO). Recent systematic studies with model silica-supported catalysts, prepared via surface organometallic chemistry (SOMC), have shown that Ti, Zr, Hf, Ta, Zn, or Ga at the surface of the support enhance the methanol formation rate and the selectivity.^24–26^ How this is achieved markedly depends on the nature of the promoter, bringing forward the importance of interfacial sites and/or alloying.^9,19,24,25,27^ The promotional effect parallels well-known “support” effects found in Cu/ZnO/Al_2_O_3_, Cu/ZrO_2_, or Cu/γ-Al_2_O_3_.^2,9,11,19,27,28^ A notorious deleterious support effect with Cu is TiO_2_, which favors the RWGS reaction due to the growth of Cu nanoparticles on TiO_2_, leaving only the support as an “effective” RWGS catalyst.^29^ Conversely, single Cu atoms and clusters display remarkable CO_2_ hydrogenation to methanol activity^3,30^ when supported on two-dimensional (2D) materials like MXenes^31^ or Mo_2_CT_*x*_.^27,28^ Beyond Cu-based catalysts, it is noteworthy that Ga is a quite universal promoter, favoring methanol synthesis across most transition metals (M = Ni, Pd, Pt, Ru, Os, Rh, Ir).^27,32–38^ This sharply contrasts with Zn, which is most (and very) effective for Cu and Au^35^ but is not as general; e.g., PtZn is very selective to RWGS while PtGa promotes methanol formation,^39^ highlighting the fine balance in driving the selectivity of CO_2_ hydrogenation reactions.

Understanding the origin and role of promoters and supports favoring methanol formation at the molecular-level remains challenging due to the complexity of these catalysts, with metal–metal and metal–metal oxide interfaces, which can be dynamic considering that hydrogen (and CO) correspond to “reducing” conditions while CO_2_ (and H_2_O) correspond to “oxidizing” conditions, in particular toward the promoter elements.^27^ Furthermore, the nature of the active sites in the industrial catalyst, Cu/ZnO/Al_2_O_3_,^11,28,40^ remains highly debated, due to the presence of CuZn alloys and CuZnO interfaces. Notably, the intrinsic dynamics between these structures,^41–43^ which prevents identifying the active site(s)/states, often leads to contradictory conclusions.^11,19,43–48^ As briefly alluded to, SOMC has enabled generation of model catalysts with tailored composition and interfaces more suitable for spectroscopic characterization, allowing the correlation of catalyst states and dynamics with the detection of reaction intermediates, via operando or in situ spectroscopy. X-ray Absorption Spectroscopy (XAS), infrared (IR) spectroscopy, and NMR spectroscopy have provided key information regarding CO_2_ hydrogenation on these tailored catalysts, which has been used in combination with computational modeling to decipher these systems at the atomic level across length scales,^49–51^ highlighting key parameters: (i) the relative stability of the catalytic phase (metallic vs oxidic) under reaction conditions,^6^ (ii) the nature of possible active sites and mechanistic investigations of the energetics of CO_2_ hydrogenation, complementing spectroscopic measurements, and (iii) evaluation of the dynamics of metal particles and phase changes under reaction conditions via ab initio molecular dynamics simulations combined with metadynamics,^52–54^ complementing X-ray absorption spectroscopy measurements. Figure 2 summarizes the goal and systems discussed in this Account and the three pillars on which it is sustained.

**Figure 2:**
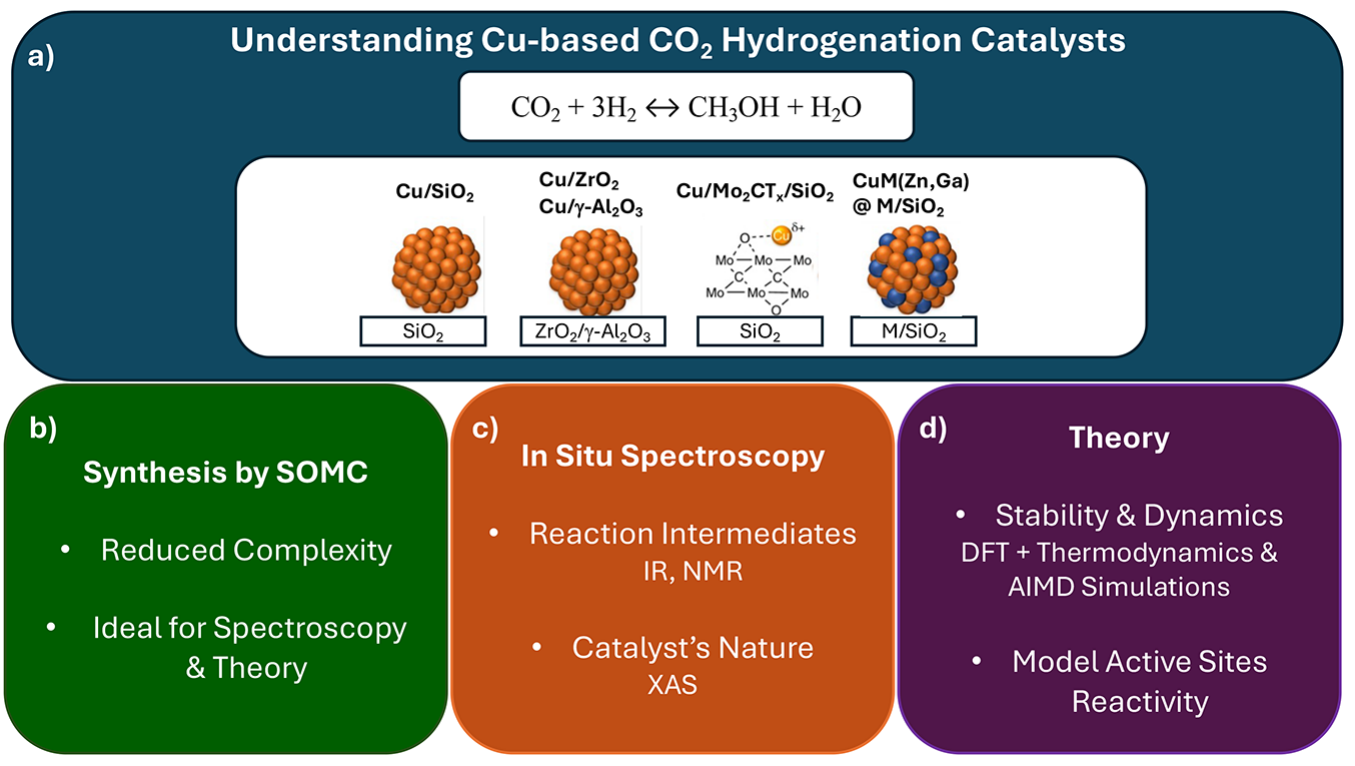
(a) The goal and systems discussed in this Account are sustained on three pillars: (b) synthesis by surface organometallic chemistry, (c) in situ spectroscopy, and (d) theory, and the respective advantages and information they provide.

In this Account, we highlight how computational methods provide further understanding of Cu-based materials in CO_2_ hydrogenation, addressing key experimental questions (structure, stability, and dynamics of Cu nanoparticles, nature of support effects, and effect of Zn and Ga promoters on structure and dynamics). Section 3 focuses on (i) the stability of Cu facets and their adsorbates under reaction conditions based on ab initio thermodynamics and (ii) the development of realistic Cu NPs supported on oxide and carbide (ZrO_2_, γ-Al_2_O_3_, MXene) models using static DFT calculations to unravel the promotional support effects. Section 4 addresses how Ga and Zn alloying tune Cu dynamics and adsorption properties and the tendency of both promoters to segregate into GaO_*x*_ and ZnO_*x*_ phases as obtained by XAS based on ab initio molecular dynamics.

## 2 Activity and Stability of Cu NPs and Oxide Promotion (ZrO_2_ and γ-Al_2_O_3_)

### 2.1 Morphology of Cu NPs and Catalytic State under CO_2_ Hydrogenation Conditions

Since Cu is the reference metal in CO_2_ hydrogenation, understanding the morphology, shape, and species adsorbed and their coverage under various conditions (reducing vs oxidizing)^55^ is crucial to comprehend more complex Cu-based catalysts.^56^ The calculated surface energies of the evaluated Cu facets in vacuum (Figure 3a) followed the trend: γ (111) < γ (100) < γ (211) < γ (110). The Wulff construction predicts the most stable shape of Cu NPs (Figure 3b), where the 111 facet predominates, with minor contributions of the 100 and 110 facets.^56^ The evaluation of adsorption of O*, H*, CO*, and CO_2_* combined with thermodynamic considerations, i.e., using ab initio atomistic thermodynamics, allows us to provide the effect on the stability of the facets under different^57^ chemical atmospheres (Figure 3c), namely, H_2_, O_2_, CO, or CO_2_ hydrogenation conditions. The surface energies of the four investigated facets as a function of the respective relevant chemical potentials are shown in Figure 2. Ab initio thermodynamics predicts a complete bulk oxidation of Cu under air exposure and reduction to metallic Cu under H_2_, which agrees with experiments. At 25 °C and 1 bar H_2_, the (111) facets become more predominant over the (100) facets compared to vacuum, with hydrogen coverages reaching 1H* ML and (1/2)­H* ML, respectively. Under CO_2_ hydrogenation conditions, the {111} family is also the most exposed, followed by a small contribution from the {100} one, where the (111) facets act as hydrogen reservoirs, covered by at least (1/2)­H* ML. Notably, no significant change in the ratio of facets is expected from the start of CO_2_ hydrogenation until the RWGS equilibrium is reached.

**Figure 3:**
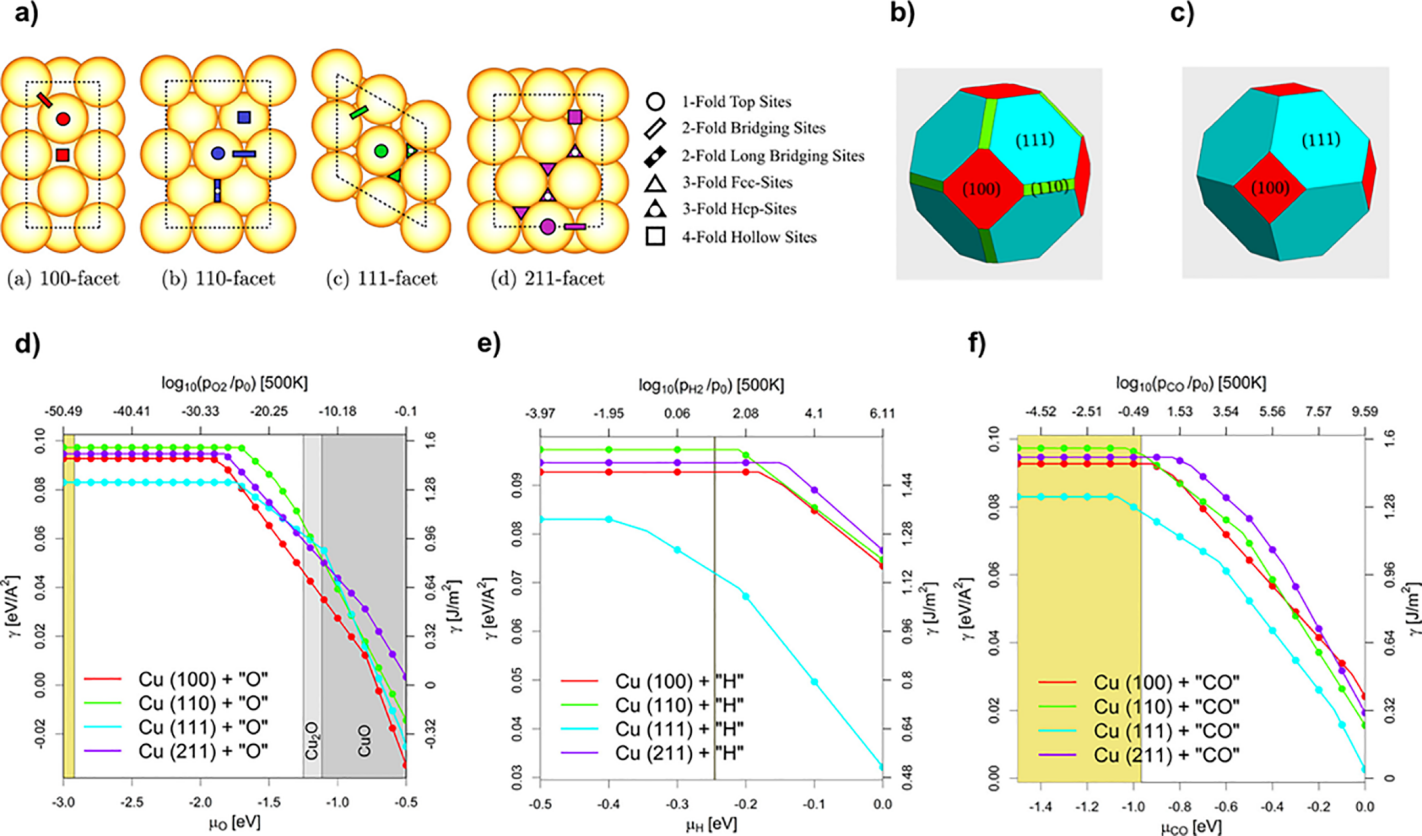
(a) Top view of the slab models used for the surface energy calculations. (b) Wulff construction for Cu particles in the vacuum. (c) Wulff construction for Cu particles under CO_2_ hydrogenation conditions. (d–f) Surface energies of the four investigated facets depending on the chemical potential of (d) oxygen (μ_O_ in eV), (e) hydrogen (μ_H_ in eV), and (f) CO (μ_CO_ in eV) and the equivalent oxygen, hydrogen, and CO partial pressures at 500 K.

### 2.2 Role of the Cu/ZrO_2_ Interface in the CO_2_ Hydrogenation to Methanol

Supporting Cu NPs on ZrO_2_ drives the selectivity of the reaction toward methanol; thus, understanding the nature of the active sites of this material is key to developing structure–activity relationships. The Cu/ZrO_2_ system displays a much higher methanol intrinsic activity and selectivity in CO_2_ hydrogenation than those of Cu/SiO_2_, as shown in Figure 4a.

**Figure 4:**
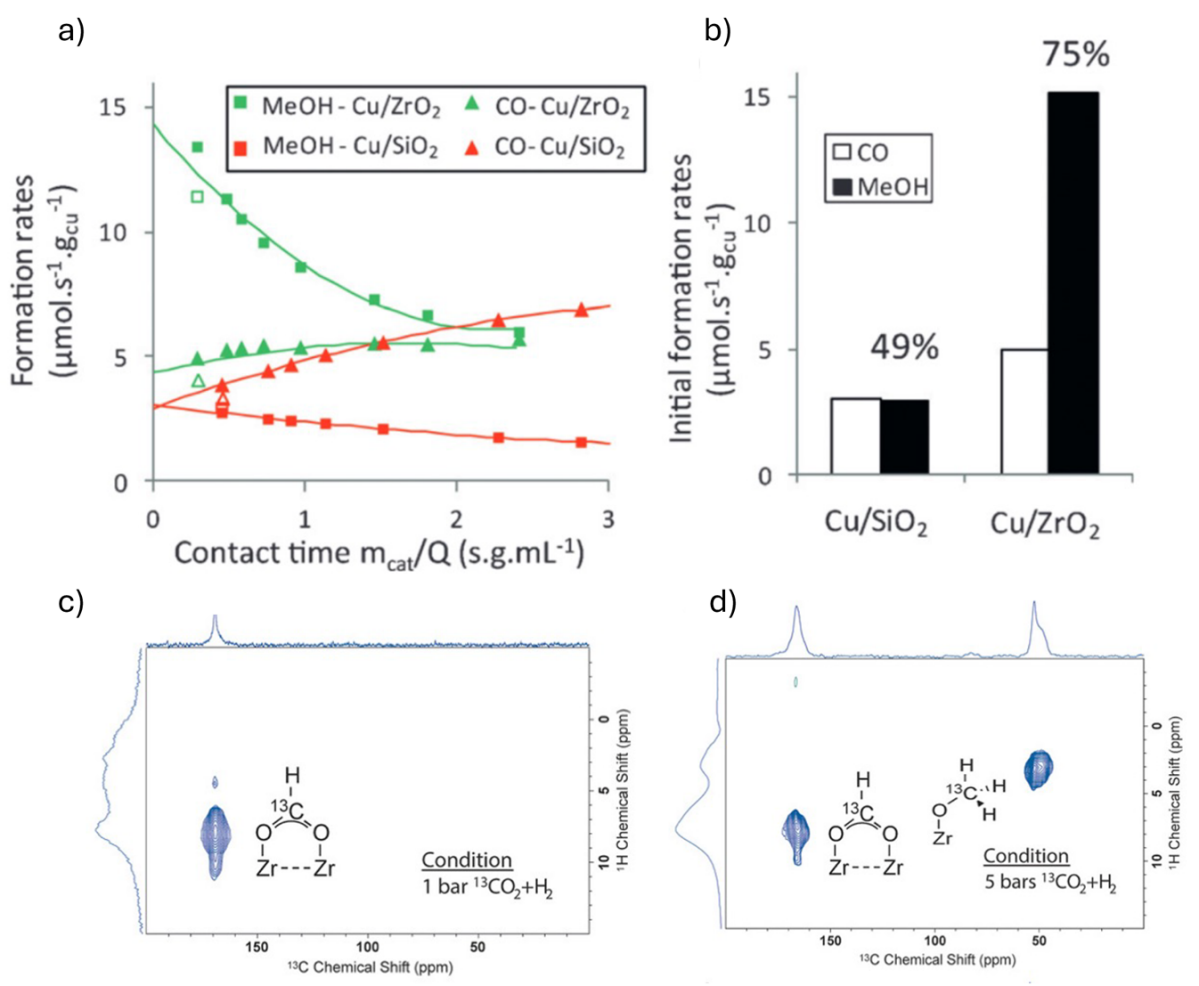
(a) Formation rates of CO and methanol as a function of contact time on Cu/ZrO_2_ and Cu/SiO_2_ measured in a flow reactor at 230 °C and 25 bar (H_2_/CO_2_ = 3:1). Unfilled symbols correspond to activity when the first data point was repeated after 40 h of reaction, showing slow deactivation. CO_2_ conversion was kept between 0.5 and 6%. (b) Extrapolated rates of formation at zero conversion. The selectivity toward methanol is also shown. (c, d) Ex situ MAS NMR ^1^H–^13^C HETCOR spectra of Cu/ZrO_2_ reacted with H_2_/^13^CO_2_ (3:1) at 230 °C for 12 h at (c) 1 bar or (d) 5 bar. External projections of the ^1^D^13^C and ^1^H spectra are applied in all spectra.

In situ DRIFTS at 230 °C under varying pressures (H_2_/CO_2_ within 1–20 bar) identifies carbonate or bicarbonate (CO_3_* and HCO_3_*) and formate species (HCOO*) on ZrO_2_, while formate and methoxy are formed on Cu/ZrO_2_, as also seen by solid-state NMR spectroscopy (see Figure 4c). The latter step occurs only at higher pressures (5 bar). It is reversible (Figure 4d), confirming that both species are key intermediates in methanol synthesis and highlighting the role of ZrO_2_ in stabilizing these intermediates. In contrast, neither species is observed on Cu/SiO_2_. The number of the active sites (0.04 per nm^–2^) can be estimated by titrating the amount of methanol molecules adsorbed on the surface formed from formate species prior to desorption using labeling experiments;^1^ this amount is comparable to the estimated density of Cu particles (0.01 per nm^–2^), suggesting a small number of active sites per Cu particle. Overall, the data support that the Cu/ZrO_2_ interface drives the selective CO_2_ hydrogenation to methanol. DFT calculations of the reaction pathway using a 38-atom Cu NP (ca. 1 nm) supported on *m*-ZrO_2_(111), despite being smaller than experimental particles (3–5 nm; i.e., having more edge and corner sites), provides a representative model to assess and highlight the role of the interface (Figure 5). While the adsorption of CO_2_ as carbonate or bicarbonate is favored on *m*-ZrO_2_(111) by −65 kJ·mol^–1^ and −70 kJ·mol^–1^, respectively, it is greatly enhanced at the Cu/ZrO_2_ interface (−179 kJ·mol^–1^), stabilized by both Cu and Zr^4+^. In sharp contrast, CO_2_ adsorbs only weakly on Cu(111) (−2 kJ·mol^–1^). In contrast, H_2_ ®activation is exoergic on the Cu(111) or Cu NP (−40 vs −50 kJ·mol^–1^, respectively), while it is endoergic by +57 kJ·mol^–1^ on *m*-ZrO_2_(111). From adsorbed CO_2_ at the Cu/ZrO_2_ interface and the hydrogen adsorbed on Cu, the formate (HCOO*), formaline (H_2_COO*, acetal-like), and methoxy species are readily formed via H-transfer (spillover) from the Cu NP to the interfacial reaction intermediates. Notably, the formaline intermediate is found to be slightly less stable than either the formate or the methoxy but connected via low-energy barriers, consistent with the reversible formate and methoxy formation according to H/D exchange, while formaline is only a transient intermediate. Methanol forms by the reaction of adjacent methoxy (CH_3_O*) and hydroxyl (OH*) species via SN_2_-like steps (52 kJ·mol^–1^), followed by its desorption, and the reaction of hydrogen with OH* to generate water. Alternative routes via CO* or COOH* are found to be less favorable, explaining the preferred formation of methoxy species.

**Figure 5:**
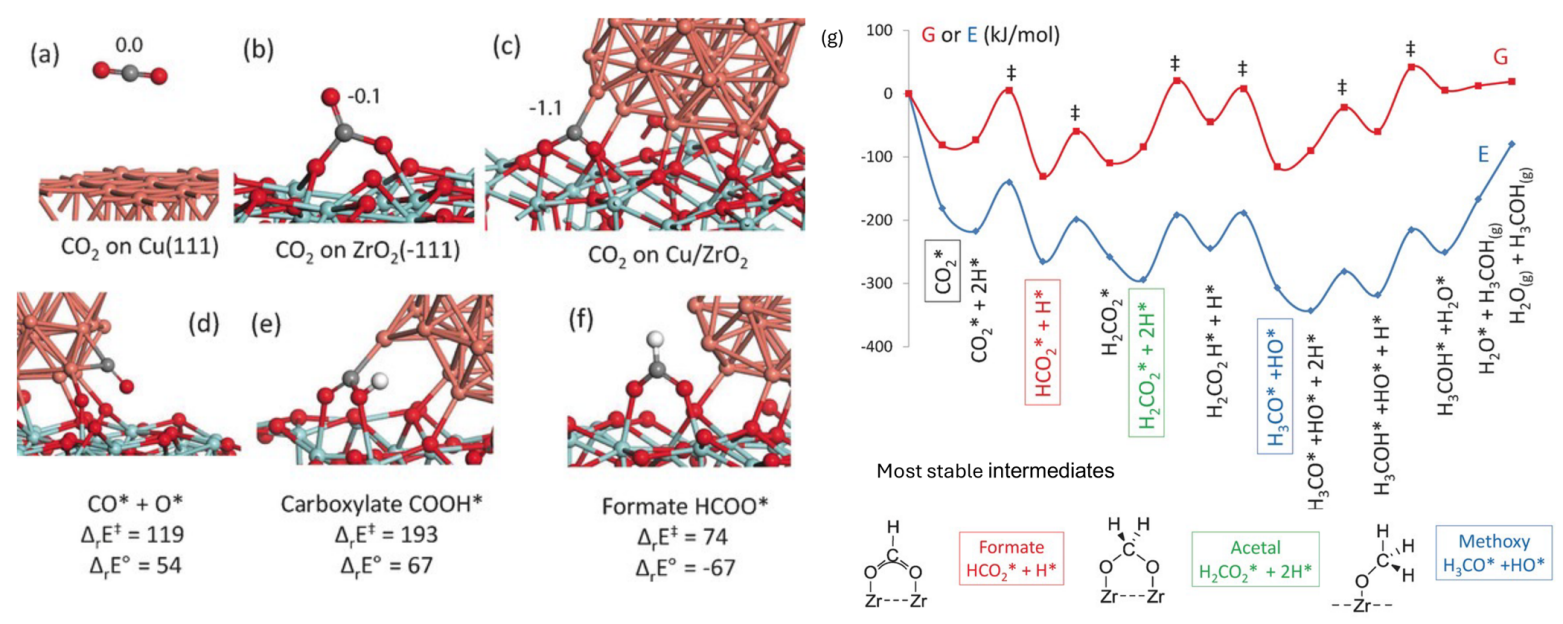
(a–c) Optimized structures of CO_2_ (a) physisorbed on Cu(111), (b) chemisorbed on *m*-ZrO_2_(111), and (c) at the interface between copper and zirconia. The Bader charge on the CO_2_ molecules is also given. (d–f) Optimized reaction intermediates at the interface between copper and zirconia: (d) CO* and O*, (e) carboxylate COOH*, and (f) formate HCOO*. Electronic energy barriers and formation energies from adsorbed CO_2_ + H_2_ are shown in kJ·mol^–1^. Atom key: zirconium (cyan), oxygen (red), copper (orange), carbon (gray), hydrogen (white). (g) Electronic and Gibbs energy profiles for CO_2_ hydrogenation to methanol at the Cu/ZrO_2_ interface at 473 K. Energetics are referenced to the Cu/ZrO_2_ model and CO_2_(g) + 3H_2_(g). The most stable intermediates in Gibbs free energy are schematically shown below.

DFT calculations suggest that the metal/oxide interface drives methanol formation from CO_2_ and H_2_ via formate and acetal reaction intermediates. The findings provide insights into the nature of active sites to guide catalyst design and the need for high pressure (5 bar) to drive the reaction.

### 2.3 Role of the Cu/Al_2_O_3_ Interface in the CO_2_ Hydrogenation to Methanol

γ-Al_2_O_3_-supported Cu NPs (3.1 ± 0.6 nm) display higher formation rates for both CO and methanol-related products, MeOH and dimethyl ether (DME), under the same CO_2_ hydrogenation conditions (230 °C and 25 bar) than Cu/SiO_2_ (Figure 6a, Cu/Al_2_O_3_). Detailed investigations by IR and solid-state NMR spectroscopy also identify formate and methoxy species as key reaction intermediates and show that the formation and subsequent decomposition of methyl formate can explain the increased formation rate of CO.

**Figure 6:**
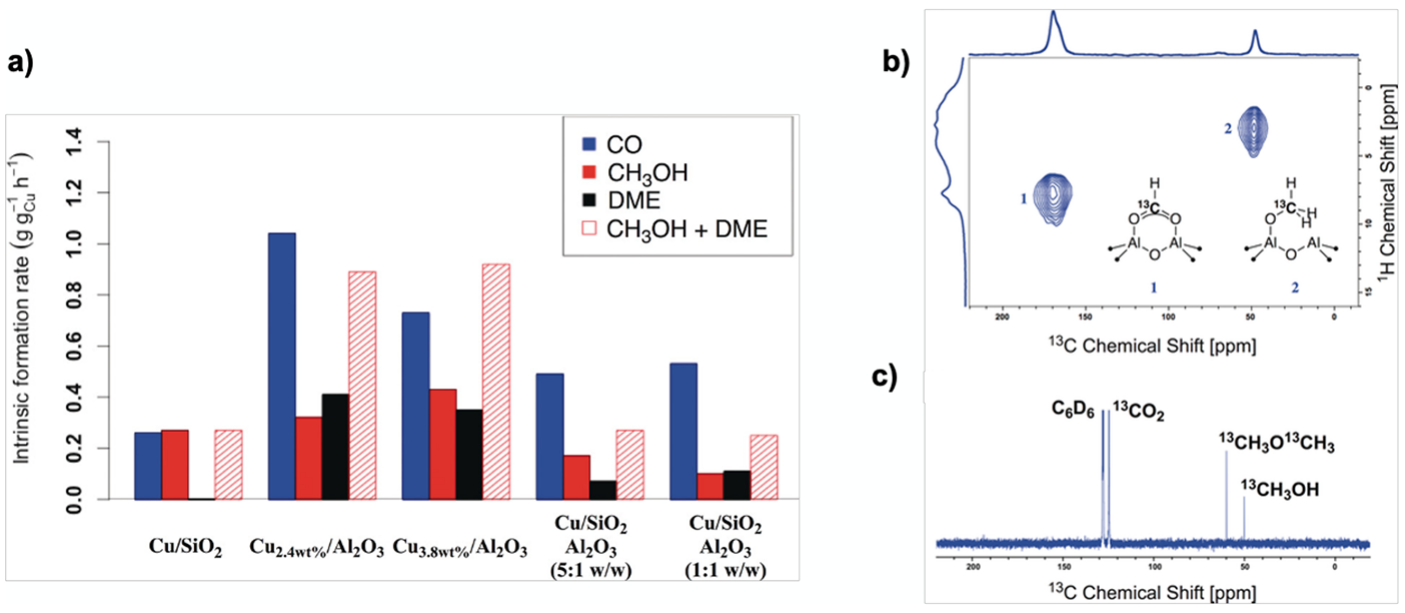
(a) Intrinsic formation rates for Cu/SiO_2_ and Cu/Al_2_O_3_, as well as physical mixtures of Cu/SiO_2_ and Al_2_O_3_ for the formation of CO (blue), CH_3_OH (red), DME (black), and overall CH_3_OH formation (CH_3_OH + DME; red-dashed). (b) Ex situ MAS NMR ^1^H–^13^C HETCOR spectra of Cu_2.4wt%_/Al_2_O_3_ reacted with ^13^CO_2_/H_2_ (1:3) at 5 bar for 12 h and 230 °C. (c) Solution ^13^C NMR of the gas phase of Cu_2.4wt%_/Al_2_O_3_ after reaction recorded in C_6_D_6_.

To probe the role of the support and interfacial sites, analogously to the Cu/ZrO_2_ system, various reaction pathways have been evaluated by DFT calculations using a Cu NP model (67 atoms, ca. 1 nm) supported on the main facet of γ-Al_2_O_3_ (110). This model presents different interfacial Al sites, namely, one tricoordinated Al site, Al­(III), and two types of tetracoordinated Al sites, Al­(IVa) and Al­(IVb) sites (Figure 7a). At 500 K, the Gibbs energies of CO_2_ adsorption on Al sites ranged from −59 to +63 kJ·mol^–1^, coordination on Al­(III) being the most stable and that on Al­(IVa) being the least stable one. This activated CO_2_ can react with H* chemisorbed on the Cu NP surface. Methanol formation proceeds via a pathway similar to that for Cu/ZrO_2_, via formate (HCOO*), acetal (CH_2_OO*), and CH_2_OOH*, followed by C–O bond cleavage, generating CH_2_O* and OH*, which hydrogenate to methanol and water, respectively. In parallel, CO_2_* can be directly converted into CO* and O* at the Cu/Al_2_O_3_ interface. The selectivity between these two pathways strongly depends on the CO_2_ coordination mode and the Al sites involved: CO_2_ on Al­(III) via the μ^2^-η^1^(C):η^1^(O) coordination mode favors CO formation, while bridged CO_2_ on Al­(IVa)–Al­(IVa) leads to formate and methanol formation. The latter site enables fast methanol formation with feasible energy barriers for key steps: acetal formation, CH_2_OOH* generation, and C–O cleavage. DME formation occurs easily on γ-Al_2_O_3_ via Lewis acidic Al sites without Cu involvement.^58^

**Figure 7:**
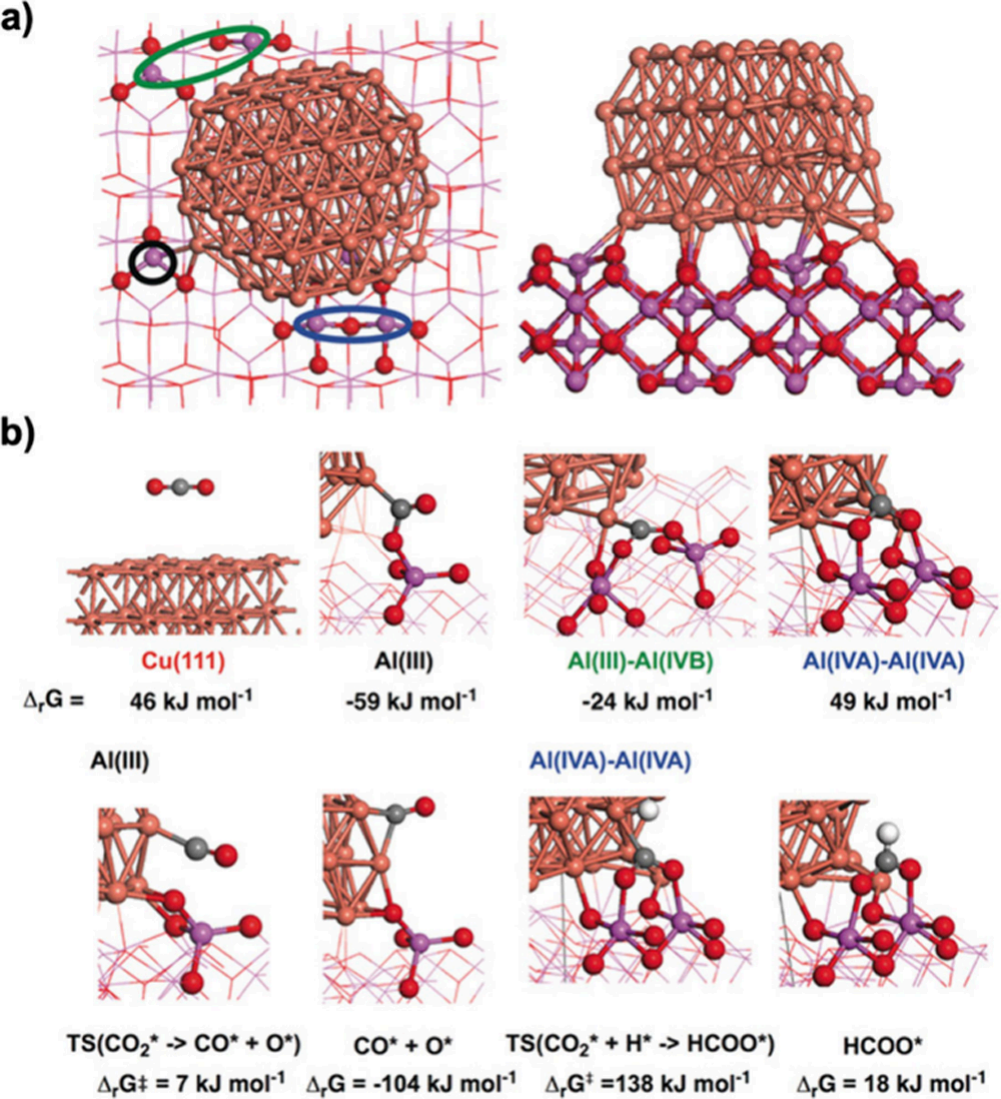
(a) Top (left) and side (right) views of the computational model consisting of the (110) facet of -Al_2_O_3_ and a Cu67 particle with the aluminum interfacial sites of the support circled for Al­(III) (black), Al­(III)–Al­(IVB) (green), and Al­(IVA)–Al­(IVA) (blue). (b) Structure and adsorption Gibbs energy (Δ_r_ *G*) calculated at 500 K in kJ·mol^–1^ for CO_2_ physisorbed on the Cu(111) facet and adsorbed on the interfacial sites: Al­(III) site (black circle), Al­(III) site nearby the Al­(IVB) site (green circle), and two adjacent Al­(IVA) sites (blue circle). Transition states for the formation of CO* + O* and HCOO* on Al­(III) and Al­(IVa)–Al­(IVa).

Compared to Cu(111) and Cu/ZrO_2_, Cu/Al_2_O_3_ enhances CH_3_OH and CO formation, where the metal–oxide interface either promotes or demotes selective catalytic paths. Again, formate and methoxy species are the most stable intermediates along the energy profile, supported by solid-state NMR and IR. Both theory and experiment support that the nature of the Cu–Al_2_O_3_ interface determines product selectivity, where Cu/γ-Al_2_O_3_ acts as a bifunctional catalyst, with Al­(IVa)–Al­(IVa) sites promoting CH_3_OH, Al­(III) favoring CO, and Al_2_O_3_ Lewis acidity driving DME and CO formation.

### 2.4 Catalytic Activity of Highly Dispersed Cu Atoms on Mo_2_CT_*x*_

Surface organometallic chemistry on a silica-supported delaminated molybdenum MXene, Mo_2_CT_*x*_, generates highly dispersed Cu, ranging from single atoms to small clusters. The catalyst displays a much higher intrinsic methanol formation rate than Cu/SiO_2_.^3^ Operando DRIFTs and solid-state NMR with labeled reactants indicate greater amounts of formate and methoxy intermediates compared to those on Cu/SiO_2_, while Auger spectroscopy and CO-DRIFTS suggest that the enhanced activity is due to a higher fraction of active Cu sites in the Cu/Mo_2_CT_*x*_ catalyst, assigned to Cu^δ+^ for the most active materials.

Computational models of Cu atoms on Mo_2_CT_*x*_ surfaces with different oxygen coverages (0.22–0.77 ML) were developed to benchmark the active site against CO-IR spectroscopy and H_2_ TPD measurements (Figure 8). A single Cu atom supported on the Mo_2_C-0.67 O ML model is the best matching model, bearing a significant cationic character, with high H_2_ adsorption and a blue shift in the CO IR stretching frequency. This cationic character opens new reactive paths via the heterolytic cleavage of H_2_, reacting with adsorbed CO_2_ and leading to formate (HCOO*) with an energy barrier equal to 103 kJ mol^–1^. Calculations also predict the high stability of methoxy (OCH_3_*) and agree with the experimental observation of this species in FTIR and NMR experiments and the feasibility of forming CO as a product via the direct cleavage of CO_2_ to CO* and O*. The most feasible route for the CO_2_ hydrogenation involves the participation of the interface and the support, cleaving HCOO* to HCO* and O* on the support, followed by their hydrogenation to CH_3_OH and H_2_O.^30^ Both CO and CH_3_OH formation paths present relatively low and comparable calculated energy barriers, while their desorption presents similar energetics.^30^ Thus, no clear selectivity is expected for either CO or CH_3_OH, in agreement with experimental observations.^3^

**Figure 8:**
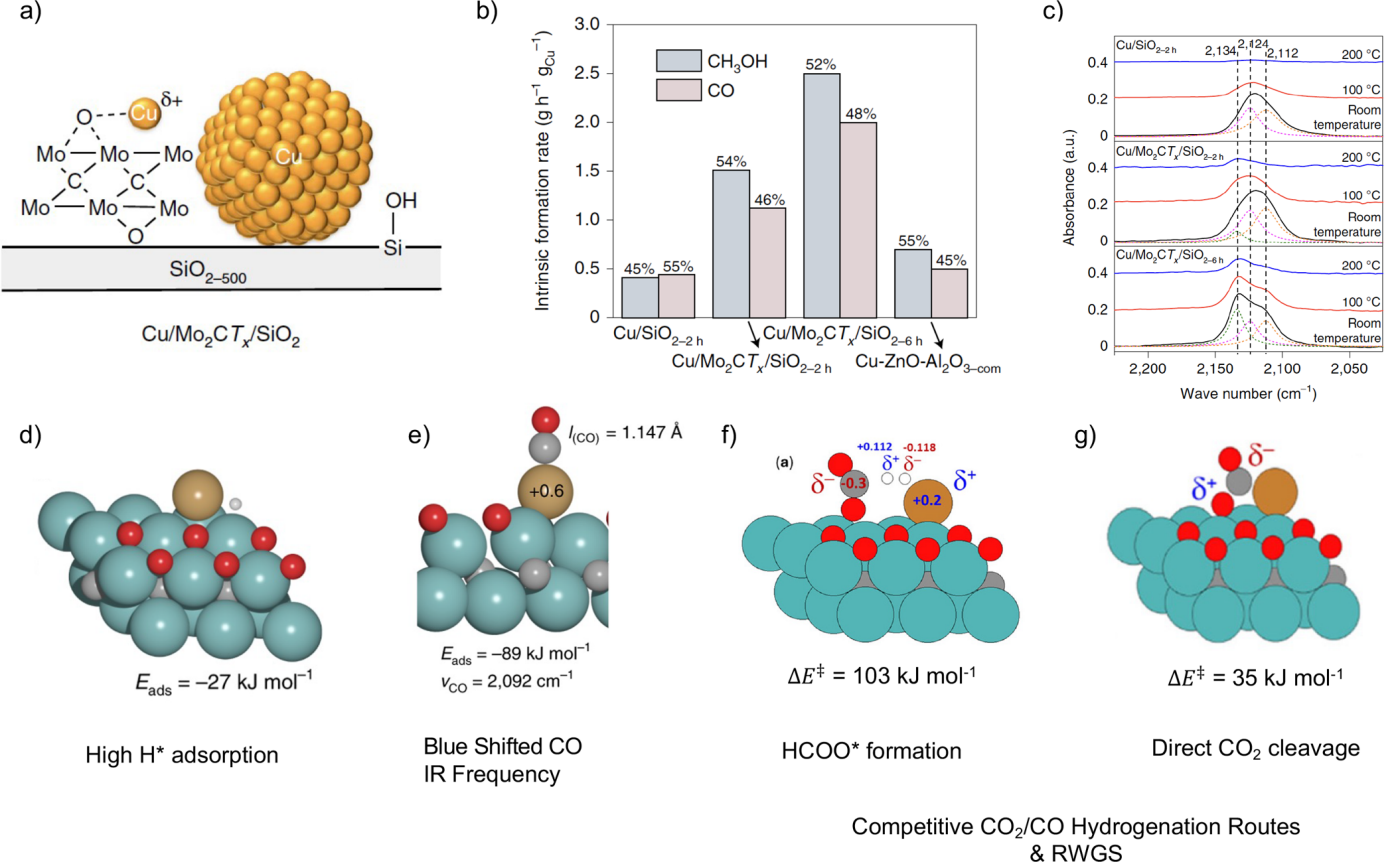
(a) Cu/Mo_2_CT_*x*_/SiO_2_–500 with highly dispersed Cu sites interacting with partially reduced Mo_2_CT_*x*_ nanosheets. (b) Comparison of intrinsic formation rates of CH_3_OH and CO for the catalysts tested (230 °C, 25 bar, H_2_/CO_2_/N_2_ = 3/1/1) obtained by extrapolation to zero conversion, together with the selectivities for CH_3_OH and CO specified above the respective bars. (c) DRIFT spectroscopy spectra after desorption of CO at different temperatures. (d) Optimized structure of H adsorbed on the Cu/2D-Mo_2_C–0.67 O ML and adsorption energy (Δ*E*). (e) Optimized structure of CO on Cu/2D-Mo_2_C–0.67 O ML, adsorption energy (Δ*E*), and computed CO IR stretching frequency. (f) Transition state corresponding to HCOO* formation and relative energy barrier (Δ*E* ^‡^). (g) Transition state corresponding to CO_2_ cleavage to CO* and O* and relative energy barrier (Δ*E* ^‡^).

These results showcase how highly dispersed Cu in the form of a Single Atom Catalyst (SAC)^60^ and stabilizing Cu^δ+^ sites can also foster methanol formation, highlighting the additional catalytic role of the support.

## 3 Ga- and Zn-Promotional Effect and Role of Alloying

A central question is how Ga- and Zn-promoters alloyed with Cu tune the catalytic activity of Cu-based catalysts. Both increase the methanol formation rate and selectivity while showing reduced inhibition effects by H_2_O and MeOH in contrast to Zr-promoted catalysts (*vide supra*). Unlike Zr systems, Ga and Zn form CuZn and CuGa alloys under hydrogen,^25,61^ while Zr only yields interfacial sites for supported Cu NPs.^1,26^ However, operando XAS spectroscopy shows that CuGa and CuZn alloys are unstable under the following CO_2_ hydrogenation conditions: CuGa fully and reversibly dealloys to Cu(0)­GaO_*x*_, while CuZn partially dealloys, generating Cu(0)­ZnO_*x*_ and CuZn/ZnO_*x*_. Notably, EXAFS points to a decrease of CuGa coordination (CN = 3–4), an increase of Cu–Cu coordination from 7 to 10–11, and a growth of Ga–O shells (CN = 4–6 at ca. 1.81 Å) for CuGa particles,^61^ while only partial dealloying is observed for CuZn, with Zn–Cu coordination decreasing from CN 3–4 to 2–3, leading to the need to include Zn–O contributions (CN = 4–5 at ca. 1.95 Å), with Cu remaining metallic (CN_Cu–Cu_ = 9–10).^25^ The reactivity of CuGa is also highly sensitive to composition since Ga-rich alloys lead to poisoning and low methanol formation rates.^62^ The questions addressed for these systems are the stability of their bulk and surface terminations, the dynamics of CuGa and CuZn particles,^63^ and the adsorption strength of relevant intermediates under CO_2_ hydrogenation conditions.^4^

### 3.1 Stability of CuZn and CuGa Bulk Alloys

CuGa and CuZn alloys form a fcc-based solid crystalline solution when introduced into the Cu lattice. CuZn alloys can adopt a richer promoter composition compared to CuGa ones,^4^ stabilizing up to a molar fraction of promoter of 0.50 (mixing energy of −2.5 eV), while for CuGa, the stabilization is limited to a Ga molar fraction of 0.25 (mixing energy of ca. −3 eV), in agreement with the experimental phase diagram.^64,65^ The most stable systems for all the evaluated facets of CuZn and CuGa alloys ((100), (111), and (110) for promoter concentrations ranging from 12.5 to 37.5% for Zn and 12.5 to 31.0% for Ga) correspond to structures with an even distribution of the alloy’s promoter (Ga, Zn).

### 3.2 Stability and Dynamics of CuGa and CuZn Nanoparticles

Ab initio molecular dynamics (AIMD) simulations of CuGa and CuZn NPs allow one to probe how particle size, simulation temperature, and alloying with Ga and Zn affect the atomic mobility and overall crystallinity of small Cu NPs.^63^ The diffusion coefficients were derived from single AIMD trajectories for each system via linear fitting of the mean-squared displacement (MSD) of atoms in the time range of 1000–2000 fs, where the MSD slope remained constant, confirming that the system reached the diffusive regime. For pure Cu NPs within 0.8–1.9 nm, at 503 K, the typical CO_2_ hydrogenation temperature (230 °C), the Cu diffusion coefficients (*D*) decrease nearly linearly with increasing particle size. Radial distribution functions show a narrow peak at 2.6 ± 0.1 Å corresponding to bulk distances, while the peaks beyond 3.0 Å are not well-defined for the smallest NPs (0.8–1.2 nm), suggesting a transition from amorphous to crystalline with increasing size. Temperature-dependent simulations of Cu_38_ confirm an Arrhenius-type mobility between 503 and 1053 K, while the particle retains crystallinity at lower temperatures. For comparison, the melting point of Cu equals 1358 K.^66^

Comparing simulations of Cu_18_Ga_20_ and Cu_18_Zn_20_ NPs vs Cu_38_, Ga_38,_ and Zn_38_ NPs (all approximately 1 nm in size) shows that Ga and Zn significantly increase Cu diffusion, with no significant differences between Ga and Zn. In contrast, the rate of diffusion increases for Ga and decreases for Zn, compared to that of the pure elements (Ga or Zn), with Zn diffusion rates being higher than Ga when alloyed with Cu (see Figure 9).^63^ Despite the difference in size between experimental (3–5 nm) and simulated particles (0.8–1.9 nm for Cu and ca. 1 nm for CuGa, CuZn, Ga, and Zn particles), both Cu particle size and alloying influence the dynamics of Cu-based catalysts, possibly affecting their catalytic behavior under operando conditions.

**Figure 9:**
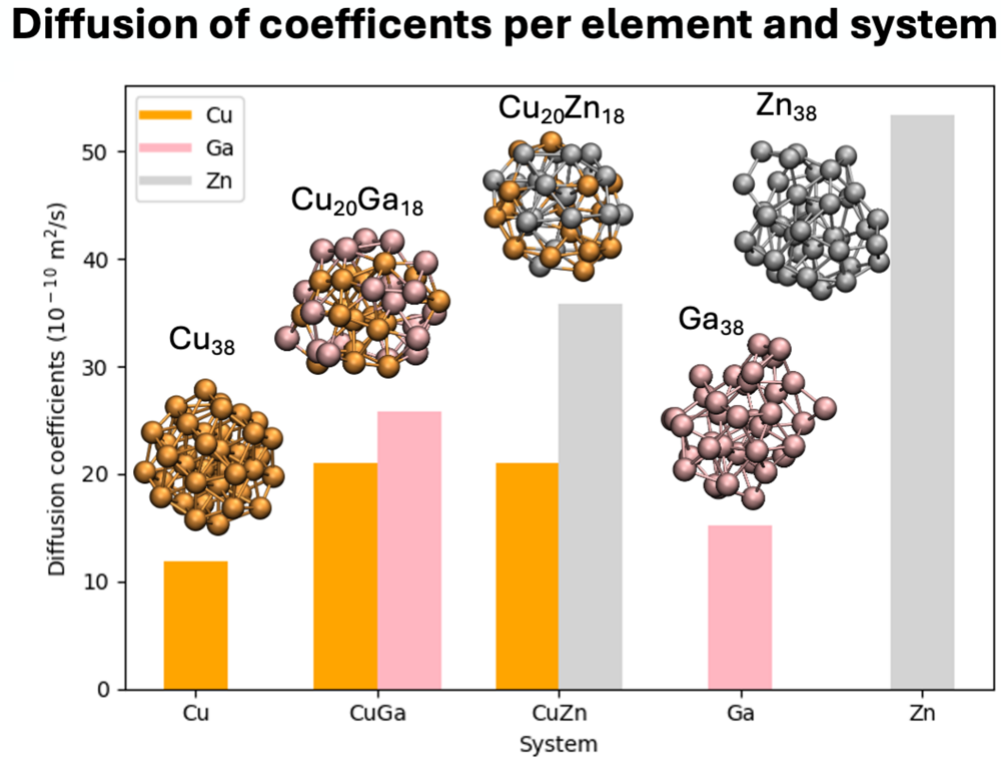
Histogram summarizing the diffusion for each of the elements composing the CuGa and CuZn particles and for pure Cu, Ga, and Zn particles with structures for each system shown as insets. Adapted from ref 63.

### 3.3 Surface Oxidation of CuGa and CuZn under CO_2_ Hydrogenation Conditions

CO_2_ hydrogenation reaction conditions can be formally viewed as an oxygen chemical potential (μ_O_) within −3.11 and −2.92 eV, equivalent to a very low O_2_ pressure, but still “oxidizing” for both Ga and Zn (Figure 10a,b). In contrast, μ_O_ under pure CO is significantly lower (ranging from −4.07 and −3.93 eV). The μ_O_ at which O* adsorption is more stable than the clean CuGa and CuZn surfaces as a function of the promoter concentration is shown in Figure 10c,d. DFT calculations show that oxygen adsorption depends strongly on Ga concentration, while CuZn is less affected, especially for a Zn concentration beyond 20%. For CuGa, the μ_O_ at which oxygen favorably adsorbs decreases linearly with Ga content up to 20% for all facets, particularly for the 110 one. For CuZn, the μ_O_ threshold of favorable oxygen adsorption decreases with Zn content up to 20% but to a lower extent than that for CuGa. Between 20 and 40%, oxygen adsorption does not depend on Zn content, while above 40% the point of oxygen adsorption only decreases for the 110 facets, favoring partial dealloying. Literature has also pointed out the stability of ZnO_3_ motifs on the Cu(111) surface.^67^

**Figure 10:**
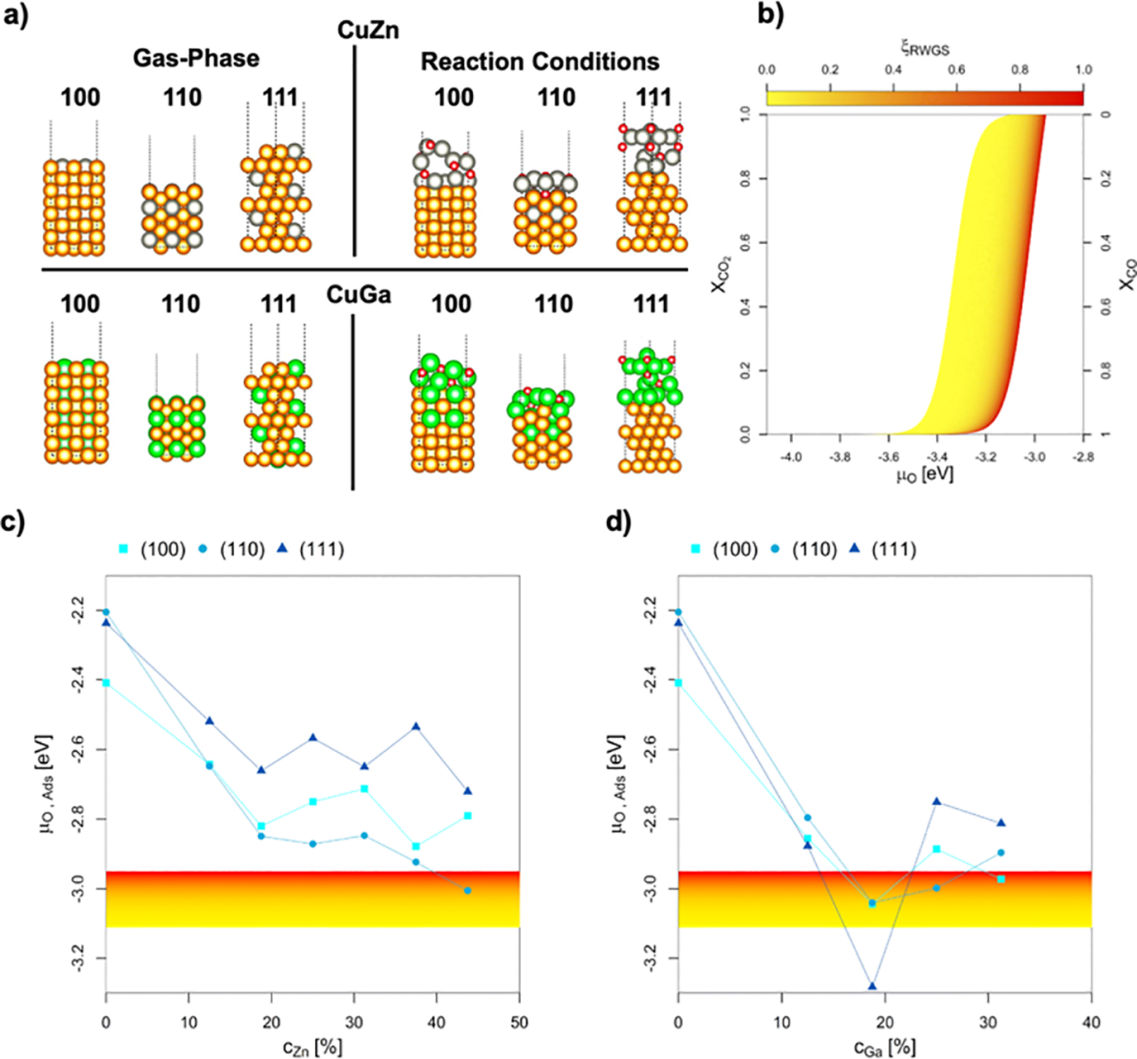
(a) The most stable Cu/Zn and Cu/Ga surfaces in the vacuum and under reaction conditions according to static DFT calculations. (b) Chemical potential of oxygen (μ_O_) under CO_*x*_ hydrogenation conditions, depending on the molar ratio of CO_2_ in the feedstock (χ_CO_2__). The yellow–red area indicates the expected oxygen chemical potential (μ_O_) under CO_2_ hydrogenation conditions (3.11 < μ_O_ < 2.92 eV). (c, d) Points of oxygen adsorption, i.e., lowest oxygen chemical potential (μ_O_) where one or multiple adsorbed O* is more stable than the corresponding clean surface for the CuZn and CuGa system, as a function of the (c) Zn (*c*_Zn_) and (d) Ga concentration (*c*_Ga_), respectively.

Overall, CuGa alloys oxidize more easily than CuZn ones under CO_2_ hydrogenation conditions: ca. 20% Ga leads to complete oxidation (Cu/GaO_*x*_), whereas CuZn undergoes only partial oxidation to Cu/ZnO_*x*_. Thus, CO_2_ hydrogenation thermodynamically favors full dealloying of CuGa to Cu/GaO_*x*_, while partial dealloying to Cu/ZnO_*x*_ is predicted for CuZn, consistent with the XAS and EXAFS data (*vide supra*).^61,62^

### 3.4 Key Sites and Stability for H* and CO* Adsorption on CuGa and CuZn Slab Models

Other key adsorbates in the CO_2_ hydrogenation are H* and CO*. Under CO/CO_2_ hydrogenation conditions, hydrogen adsorption is only favorable on the 3-fold adsorption sites composed of three Cu atoms of the (111) facets of CuGa and CuZn,^4^ resembling the most stable adsorption site for H* on a Cu(111) facet.^56^ Accordingly, hydrogen adsorption is likely only favorable for Ga/Zn concentrations of up to 25%, where such sites are present. CO adsorption behaves similarly to the hydrogen adsorption; the 3-fold sites of the (111) termination composed of Cu atoms were the only ones favored, since Zn and Ga disfavor its adsorption, being only favored if the promoter concentration is low (<25%). Concerning CO_2_ adsorption, although not evaluated, it is likely favored on Cu/MO_*x*_ (M = Ga, Zn) interfaces, based on the high stability of CO_2_ on Cu/ZrO_2_ and Cu/Al_2_O_3_ interfaces (*vide supra*).

## 4 Conclusions and Outlook

Tailored heterogeneous catalysts prepared via surface organometallic chemistry (SOMC) have highlighted the role of metal–metal oxide interfaces and alloying in CO_2_ hydrogenation to methanol. For Cu-based catalysts, elements like Zn and Ga, present on oxidic supports with Lewis acid surface sites and on catalyst formulations alloyed with Cu, typically improve the catalyst performances regarding activity and methanol selectivity. In all cases, the improvements have been ascribed to the presence of Cu(0)–metal oxide interfaces. This perspective clarifies the role of the gas phase composition and interfaces in driving the selectivity toward methanol synthesis vs the RWGS reaction, and why Ga and Zn, while both acting as promoters, show similar yet different behaviors.

Ab initio thermodynamics indicate that the 111 facet dominates Cu NPs, acting as a hydrogen reservoir under CO_2_ reaction conditions. DFT calculations on model Cu NPs supported on ZrO_2_ suggest that Lewis acid Zr^4+^ sites at the interface with Cu particles significantly enhance the methanol formation rate and selectivity by favorably adsorbing CO_2_ and enabling H-transfer from H_2_ activated on Cu to spillover to CO_2_* to form key reaction intermediates such as formate and methoxy, detected via spectroscopic measurements. A similar pathway is found for alumina-supported Cu NPs. However, not all Al^III^ Lewis acid sites equally foster methanol formation, since the low-coordinated and most acidic and reactive Al sites also promote the RWGS reaction. Notably, with highly dispersed Cu sites supported on Mo_2_CT_*x*_ materials, models benchmarked against spectroscopic measurements show that a cationic single Cu site, as in a Single Atom Catalyst (SAC),^60^ opens alternative reaction pathways for methanol synthesis via H_2_ heterolytic cleavage, which subsequently reacts with CO_2_ to generate methoxy surface species.

Zn and Ga promoters readily alloy with Cu under reducing conditions (H_2_) and foster methanol synthesis by forming Cu(0)–ZnO_*x*_ or Cu(0)–GaO_*x*_ interfaces under CO_2_ hydrogenation conditions, as found with Cu NPs dispersed on Lewis acidic supports, yielding Cu(0)/ZrO_2_ or Cu(0)/Al_2_O_3_ interfacial sites. First-principles calculations on slab models indicate that Ga oxidizes more than Zn. Furthermore, AIMD simulations of ca. 1 nm particles found higher Cu and Ga diffusion in CuGa than for monometallic analogs, while in CuZn, Cu diffusion increases and Zn diffusion decreases. However, Zn remains far more mobile than Ga. The lower mobility of Ga and its higher oxidation tendency^100,101^ can hint at why higher Ga contents of Cu lead to poisoning,^102^ as suggested by experimental and very recent AIMD results,^103^ and why CuZn alloys are more resilient to a broader range of reaction conditions.^62,103–105,106^ The high tendency of Ga to oxidize also parallels what has been found for PdGa nanoparticles (NPs) supported on Ga­(III)-doped under different chemical atmospheres, including CO_2_ hydrogenation conditions.^30^

Despite the progress described in this Account, various challenges remain to understand these materials via further atomistic simulations and heterogeneous catalysts: (i) More realistic models are needed: typical systems simulated at DFT/AIMD level correspond to slab models/small metal particles (ca. 1 nm), differing significantly from experimental systems composed by NPs within 3–5 nm. Support effects have so far been neglected; for instance, for SiO_2_, available amorphous models^68^ or recently developed ones^69^ should be considered. (ii) A more extensive evaluation of the stability of the materials’ phase space under reaction conditions is required, besides CuZnO_*x*_/CuGaO_*x*_ formation; for instance, ZnO_*x*_H_*y*_ has been found to arise as a stable species for CuZn materials under CO_2_ hydrogenation conditions.^70^ (iii) Longer MD simulations, which have been so far limited to tenths of ps, are needed. (iv) The energetics of CO_2_ hydrogenation should be evaluated on the most relevant active sites, most likely metal/oxide interfaces, further considering the interplay of reactive events.

Tackling this additional complexity is impossible via the “classical” computational approach. Here, the development of interatomic machine learning potentials for heterogeneous catalysts^71^ and the advent of foundational models in chemistry^72^ can fill gaps concerning model size and increase complexity, as well as the length of the dynamic simulations up to nanoseconds. Global optimization techniques^73–76^ can also foster the evaluation of the materials phase space under reaction conditions and sample the distribution of active sites.^77,78^ Enhanced sampling techniques,^79–81^ as used for related systems^82^ and other heterogeneous catalysts,^83–85^ will also be essential to accelerate the description of rare events, evaluate reaction networks more extensively before using microkinetic modeling to map catalytic activity,^86^ and assess dynamic changes of the catalytic materials under operando conditions.^87^ All of these aspects will guide computational heterogeneous catalysis research in the coming years to further enhance the dialogue between theory and experiment.
